# Infant contact in day-care centres in Vietnam: A cross-sectional study to understand infant infection risk

**DOI:** 10.12688/wellcomeopenres.15238.2

**Published:** 2019-06-11

**Authors:** Michiko Toizumi, Lay-Myint Yoshida, Motoi Suzuki, Hien Anh Thi Nguyen, Amy Pinsent, Duc Anh Dang, Stefan Flasche

**Affiliations:** 1 Department of Pediatric Infectious Diseases, Institute of Tropical Medicine, Nagasaki University, Nagasaki, Japan; 2 Department of Global Health, School of Tropical Medicine and Global Health, Nagasaki University, Nagasaki, Japan; 3 Department of Clinical Medicine, Institute of Tropical Medicine, Nagasaki University, Nagasaki, Japan; 4 Infectious Disease Surveillance Center, The National Institute of Infectious Diseases, Tokyo, Japan; 5 Department of Bacteriology, National Institute of Hygiene and Epidemiology, Hanoi, Vietnam; 6 Department of Infectious Disease Epidemiology, Faculty of Epidemiology and Population Health, London School of Hygiene and Tropical Medicine, London, UK

**Keywords:** infant, skin-to-skin contact, social behaviour, child day care, Streptococcus pneumoniae

## Abstract

**Background**: Infant contact information (skin-to-skin contact between infants and others) is important to understand *Streptococcus pneumoniae* transmission patterns. A few studies have investigated infant contact patterns by asking the mother/guardian to record all contacts a child makes in one day. However, this approach does not capture contact behaviour in day-care. Our study describes the frequency and nature of physical contacts of infants in day-care to understand infant infection risk in day-care in Nha Trang, Vietnam.

**Methods**: This cross-sectional study enrolled infants aged <12 months, attending 10 randomly selected day-care centres in Nha Trang. Physical contacts of each infant for one day at the day-care centre were observed. The mean number of infants’ contacts and factors associated with contact numbers were assessed using negative binomial regression.

**Results**: In total 14 infants, aged 6-11 months, were enrolled, and a total of 96 contacts were recorded. The mean number of contacts an infant made in one day was 6.9. Infants who walked independently (age-adjusted rate ratio 1.68, 95% confidence interval 1.06-2.68) and those cared for in a larger group (1.99, 1.42-2.79) had more contacts at day-care. About 50% of infants made contact with at least one person from a commune different from the infant’s, and 50% made contact with at least one other infant at day-care.

**Conclusion**: This study found that day-care attendance may be one factor that increases contact rates of infants in Nha Trang and diversifies those contacts in terms of age and geographical spread. In this study, day-care attendance not only increased contact rates beyond those usually experienced by young children cared for at home but specifically increased contact rates with other children and adults from other communes. Day-care may play a key role in the transmission of respiratory pathogens like *Streptococcus pneumoniae* to infants.

## Introduction

*Streptococcus pneumoniae* can cause otitis media, meningitis, sepsis, and pneumonia. Young children and the elderly are most at risk for contracting these diseases ^1^. While pneumococcal conjugate vaccines (PCVs) have substantially reduced the burden of pneumococcal disease, their high price and the current World Health Organization recommendation for routine infant immunisation schedules of at least three doses introduce a substantial financial burden. To mitigate some of these costs and enhance affordability of PCVs, reduced dose schedules have been proposed that may sustain the pronounced herd effects of a mature PCV programme ^2^. However, reducing the number of doses in the priming schedule during infancy will leave infants with inferior direct protection compared to a three-dose prime and boost schedule ^3^.

To better assess the potential risks of a reduced dose of PCV schedule, we need to understand where infants contract pneumococci and from whom, as well as whether those groups are likely vaccine protected and hence offer indirect protection to infants. These considerations include both questions on what age groups infect infants and what proportion of transmission is local as opposed to coming from potentially unvaccinated populations across commune, city, or country borders.

Several age-stratified contact studies have been performed ^4– 6^, yet the contact patterns of infants remain poorly described, not only because we cannot ask them directly but also because mothers do not have an overview of their contact during a whole day when they are in day-care ^7, 8^. A study in the UK ^8^, one in Turkey ^7^, and another in Nha Trang, 2017 (Kovacs, personal communication) investigated contact patterns focusing on infants by asking the mother/guardian to record all the contacts in one day. However, their methods were not effective to capture the actual contact behaviour in the day-care setting because mothers/guardians often do not know what occurs daily in that setting. There are no previous studies targeting infant contact in day-care.

This study aimed to describe the frequency and nature of infants’ physical contacts, as close interpersonal contacts relevant to the transmission of *Streptococcus pneumoniae* ^9, 10^, in the day-care setting to aid our understanding of infant infection risk stemming from these settings in Nha Trang, Vietnam.

## Methods

### Study design and area

This cross-sectional study was conducted in November 2018 within the city boundaries of Nha Trang, south central Vietnam, which is the capital of Khanh Hoa province and has about 400,000 inhabitants. Nha Trang is a tropical coastal city with a mix of high income through tourism and poorer rural areas ^11^. Mean number of family members in a household was 5.4 (standard deviation 2.1) in a community survey targeting children under two, in Nha Trang, 2016 ^12^. In that survey, 6% of infants aged <12 months and 46% of children aged 12–23 months were attending day-care centres or nurseries.

### Selection and enrolment of participants

Infants aged less than 12 months from 10 day-care centres in Nha Trang and living in Nha Trang were eligible for enrolment. We excluded infants who were absent from the day-care centres on the observation day and those whose parent and/or legal guardian did not consent to the study. One to three infants from each day-care centre were enrolled because we felt that three was the maximum number of infants an observer could closely observe at the same once. Communes, day-care centres, and infants for the study were selected as follows: Khanh Hoa Health Service and commune health centres in Nha Trang provided a list with known day-care centres that are attended by infants. We randomly selected 10 Nha Trang communes with at least one infant attending a day-care centre under the following criteria that ensured a spread of day-care centres across the whole study area: 1) for each of the five arms of an ongoing cluster randomised PCV trial in the Nha Trang area, in which all 27 communes in Nha Trang were randomly divided into five arms ^12^, 2) randomly select one rural and one urban commune with at least one infant attending a day-care centre, 3) randomly select one day-care centre per selected commune, and 4) randomly select up to three infants per day-care centre for enrolment. In Vietnam, a commune is the smallest municipal administrative unit, and each commune has one commune health centre providing basic healthcare services for the community including vaccination. Nha Trang City comprises 27 communes.

### Written consent

Written informed consent from the infants’ parents and/or legal guardians and from the day-care centres was obtained prior to participation in this study.

### Data collection and entry

Trained field workers from Khanh Hoa Health Service (KHHS) observed and recorded the physical (skin-to-skin) contacts of each infant for one day at the day-care centre (from arriving to leaving) using a contact record form which was developed in line with previously conducted contact surveys (see extended data: Form 1 ^13^) ^4, 7, 8^. First, the observers chronologically noted each physical contact with the name of the contact person and the precisely measured time of the contact. Secondly, they added up the times of the contacts by the contact person and recorded the contacts using the contact record. Additionally, background information on infant age, gender, residency, day-care attendance history/frequency, physical ability (holds one’s head up, rolls over, crawls, sits up on one’s own, pulls oneself up, walks), and how the infant takes a nap in day-care (in a cot alone/with other children, on the floor alone/with other children) was collected as well as the age, gender, and residency of the contacts. Information on duration of contact (5 min, 5–59 min, more than 1 hour), contact frequency (daily/almost daily, once/twice a week, once/twice a month, less than once a month, never met before) was also recorded. Information on location and size of the day-care centres was collected as well (see extended data: Form 2 ^13^).

### Sample size calculation

Assuming a mean of 5 contacts per infant based on previous studies which recorded mean number of contacts in a day as 4.3 to 6.68 (Kovacs, personal communications; ^7, 8^) and that the number of contacts follows a t-distribution, a precision of +/-1.5 contacts at the 95% confidence level was expected to be obtained by enrolling at least 10 infants. Hence, we targeted 10 to 30 infants, 1 to 3 infants from each of 10 day-care centres, for this study.

### Statistical analysis

Infant physical contact patterns were described by tabulation. The mean number of contacts of infants was compared in each of the characteristics of infants and day-care centres using negative binomial regression analysis. Estimated coefficients were exponentiated and transformed to rate ratios (RR) assuming all the infants stayed in day-care for the same length of time. Crude RR was adjusted by age group (6–9 vs 10–11 months) with confidence intervals (CIs) adjusted for the clustering of day-care centres (communes) using robust standard errors. Statistical analyses were conducted using STATA version 14.0 (StataCorp LLC, TX, USA).

### Ethical approval

This study was approved by the Institutional Review Boards of the London School of Hygiene and Tropical Medicine (LSHTM) (LSHTM ethics ref. 15892) and the National Institute of Hygiene and Epidemiology (NIHE), Hanoi (IRB-VN01057-04.1/2018).

## Results

### Enrolment of participants

We selected one urban and one rural commune each from four arms of the ongoing trial ^12^ and two rural communes from the remaining arm because no urban commune in that arm had any infants registered as attending day-care centres. Each of the selected 10 day-care centres had between 1 and 3 infants in regular attendance. In total, 18 infants from 10 day-care centres were eligible for the study. Four of those infants did not attend the day-care centre on the day of the survey and were thus not enrolled in the study. Finally, three infants from one day-care centre, two each from two day-care centres, and one each from the remaining seven centres (i.e. 14 infants, in total, from 10 day-care centres) were enrolled for the study.

### Characteristics of the participants

A total of 14 infants, 9 boys and 5 girls, aged between 6 and 11 months, were observed at the 10 day-care centres on a designated day in November 2018 ( Table 1 and Underlying data ^13^). The days were chosen at the observers’ convenience. All were weekdays; seven observations were conducted on Tuesdays, five on Wednesdays, and two on Thursdays. Nine and five infants were living in rural and urban communes, respectively. Seven infants were living in different communes from those of the day-care centres. They had started attending day-care when they were 3–11 months old. They attended the day-care centres 2–7 times per week and stayed there for 495–670 minutes on the survey day (mean and standard deviation [SD] were 587.8 and 46.5 minutes, respectively). The monthly fees for the day-care centres were between 480,000 and 2,500,000 VND, and the mean was 1,591,429 (SD 567,950) VND or approximately 68.7 USD.

**Table 1.** Effect of each characteristic on number of contacts per infant at day-care, estimated using negative binomial regression model.

Characteristics	Number	Mean (SD)	Rate ratio	Age adjusted rate ratio *
Total	14	6.9 (3.2)		
Demographics				
Gender				
Male	9	6.9 (2.9)	reference	reference
Female	5	6.8 (4.1)	0.99 (0.59-1.64)	0.95 (0.55-1.65)
Age (months)				
6–9 months	4	5.5 (1.7)	reference	
10–11 months	10	7.4 (3.6)	1.35 (0.78-2.31)	
Infant's activity				
Hold his/her head up and roll over				
Yes	14	6.9 (3.2)	NA	NA
No	0	-	-	-
Crawl				
Yes	13	6.8 (3.4)	0.98 (0.38-2.49)	0.71 (0.51-1)
No	1	7.0	reference	reference
Sit up on his/her own				
Yes	13	6.8 (3.4)	0.98 (0.38-2.49)	0.71 (0.51-1)
No	1	7.0	reference	reference
Pull himself/herself up				
Yes	12	6.9 (3.5)	1.06 (0.53-2.15)	0.69 (0.42-1.14)
No	2	6.5 (0.7)	reference	reference
Walk				
Yes	3	10.3 (2.9)	1.75 (1.14-2.68)	1.68 (1.06-2.68)
No	11	5.9 (2.7)	reference	reference
Sleep				
in a cot/hammock	10	7.7 (2.5)	1.62 (0.95-2.76)	1.65 (0.72-3.76)
on the floor	4	4.8 (4.3)	reference	reference
Socioeconomic status				
Fee for day-care (VND per month)				
480,000–1,700,000	8	8.6 (2.7)	1.92 (1.23-2.99)	1.88 (1.13-3.14)
2,000,000–2,500,000	6	4.5 (2.3)	reference	reference
Infant’s residence				
Rural	9	7.0 (3.1)	0.94 (0.57-1.57)	1.01 (0.55-1.84)
Urban	5	6.6 (3.8)	reference	reference
Day-care is outside of infant’s commune				
Yes	7	6.6 (3.0)	0.92 (0.57-1.49)	0.99 (0.6-1.64)
No	7	7.1 (3.6)	reference	reference
Frequency of attending day-care				
Less (2–5 times/week)	2	9.0 (4.2)	1.38 (0.75-2.56)	1.29 (0.72-2.30)
More (6–7 times/week)	12	6.5 (3.1)	reference	reference
Day-care centre				
Number of children in the day-care				
3–12	7	7.0 (2.2)	reference	reference
16–76	7	6.7 (4.2)	0.96 (0.59-1.56)	0.96 (0.57-1.62)
Number of childcare persons in the day-care				
2–3	8	7.0 (2.1)	reference	reference
4–12	6	6.7 (4.6)	0.95 (0.58-1.56)	0.89 (0.48-1.63)
Size of the day-care center				
30–45 m^2	8	7.0 (2.1)	reference	reference
65–232 m^2	6	6.7 (4.6)	0.95 (0.58-1.56)	0.89 (0.48-1.63)
Number of children in infant’s group at day-care				
1–6	9	5.0 (1.9)	reference	reference
9–16	5	10.2 (2.2)	2.04 (1.37-3.05)	1.99 (1.42-2.79)
Number of childcare persons in the infant’s group at day-care				
1–2	12	6.9 (3.5)	reference	reference
3	2	6.5 (0.7)	0.94 (0.47-1.9)	0.85 (0.59-1.23)
Size of the infant’s room at day- care				
25–36 m^2	8	6.6 (3.9)	reference	reference
40–55 m^2	6	7.2 (2.4)	1.08 (0.67-1.76)	1.02 (0.61-1.72)
Place of day-care centre				
Rural	10	6.9 (3.0)	1.02 (0.6-1.75)	1.03 (0.55-1.93)
Urban	4	6.8 (4.3)	reference	reference

*Rate ratios adjusted by age group considering clustering in each day-care centre (commune).

All infants could hold their head up and roll over. Three infants could walk alone, nine could not walk but could pull themselves up, one could not pull himself up but could crawl and sit up on his own, and one could not crawl and sit up on his own but could roll over. Regarding sleeping arrangements at the day-care centres, ten infants slept on a cot or hammock alone, and four slept on the floor alone.

### Characteristics of the day-care centres

A total of 10 day-care centres in Nha Trang were enrolled for this study. Six were in rural and four were in urban communes. The day-care centres accommodated between 5 and 76 children, including 1 to 3 infants aged between 6 and 11 months, 2 to 75 children aged between 12 and 60 months, and 2 to 12 childcare staff members. The size of the day-care centres was between 30 and 232 square metre (median and IQR were 55 and 95, respectively).

### Number of contacts per infant

The number of contacts an infant made in one day ranged between 1 and 12, and the mean was 6.9 (SD = 3.2). This SD yielded a precision of +/-1.8 contacts at the 95% confidence level. Infants who could walk independently (age-adjusted rate ratio [aaRR] 1.68, 95%CI 1.06-2.68), whose parents were paying lower fees for the day-care (aaRR 1.88, 95%CI 1.13-3.14), and who were cared for in a group with more children at day-care (aaRR 1.99, 95%CI 1.42-2.79) on average had more contacts at day-care ( Table 1). If further adjusted for the number of children in the participating infant’s group, the RR of infants paying lower monthly day-care fees was 1.33 (95%CI 0.86-2.07). Infants’ age, gender, and residence (rural/urban), number of childcare persons in a day-care centre/the infant’s group, size of a day-care centre/a room for the infant, day-care centre’s location (rural/urban), frequency of infant’s attending day-care, and how the infant takes a nap were not associated with increased contact rates.

### Characteristics of contacts

A total of 96 contacts across the 14 infants were observed at the day-care centres ( Table 2). Of the contacts, 63% occurred with other children cared for at the day-care centres, and 31% were with childcare staff working there. The remaining contacts were with the family of childcare personnel (n = 3), other children’s parents (n = 1), and one mother and one father who visited her/his child in the middle of day-care (n = 2). A disproportionately high proportion of contacts were female (67% vs 33%), largely because all childcare personnel included in this study were female. The gender ratio among child-only contacts was well balanced. It also could be seen in less assortative gender contacts among childcare personnel. Most contacts were reported to have occurred daily or almost daily (84%), and often lasted shorter than five minutes in duration (46%). Contact with other children tended to be short; 68% were shorter than five minutes. On the other hand, contacts with childcare personnel were longer, with 97% lasting longer than five minutes and 43% lasting more than one hour.

**Table 2.** Characteristics of contacts at day-care centres (n=96).

Characteristics	Total (n=96) Number (%)	Child cared (n=60) Number (%)	Childcare worker (n=30) Number (%)
Working/cared at the day-care centre			
Child cared	60 (62.5)		
Childcare worker	30 (31.3)		
Other	6 (6.3)		
Gender			
Male	32 (33.0)	29 (48.3)	0 (0.0)
Female	65 (67.0)	31 (51.7)	30 (100.0)
Assortativeness by gender			
Same gender as the infant’s	39 (40.6)	26 (43.3)	9 (30.0)
Different gender from the infant’s	57 (59.4)	34 (56.7)	21 (70.0)
Age group (years)			
0–5	60 (62.5)	60 (100.0)	0 (0.0)
6–10	1 (1.0)	0 (0.0)	0 (0.0)
11–15	0 (0.0)	0 (0.0)	0 (0.0)
16–20	1 (1.0)	0 (0.0)	1 (3.3)
21–25	6 (6.3)	0 (0.0)	6 (20.0)
26–30	8 (8.3)	0 (0.0)	5 (16.7)
31–35	4 (4.2)	0 (0.0)	3 (10.0)
36–40	6 (6.3)	0 (0.0)	6 (20.0)
41–45	2 (2.1)	0 (0.0)	2 (6.7)
46–50	5 (5.2)	0 (0.0)	5 (16.7)
51–55	2 (2.1)	0 (0.0)	2 (6.7)
56–60	0 (0.0)	0 (0.0)	0 (0.0)
61–65	1 (1.0)	0 (0.0)	0 (0.0)
Contact’s residence			
Rural	69 (71.9)	45 (75.0)	22 (73.3)
Urban	22 (22.9)	15 (25.0)	3 (10.0)
Outside of Nha Trang	5 (5.2)	0 (0.0)	5 (16.7)
Contact’s residence different from the infant’s			
Yes	43 (44.8)	24 (40.0)	16 (53.3)
No	53 (55.2)	36 (60.0)	14 (46.7)
Contact duration			
<5 minutes	44 (45.8)	41 (68.3)	1 (3.3)
6 min – 1 hour	37 (38.5)	18 (30.0)	16 (53.3)
>1 hour	15 (15.6)	1 (1.7)	13 (43.3)
Contact frequency			
Daily or almost daily	81 (84.4)	49 (81.7)	28 (93.3)
Once or twice a week	13 (13.5)	10 (16.7)	2 (6.7)
Once or twice a month	1 (1.0)	1 (1.7)	0 (0.0)
Less than once a month	0 (0.0)	0 (0.0)	0 (0.0)
Never met before	1 (1.0)	0 (0.0)	0 (0.0)

The age of contacts ranged from 0 to 65 years, with a median contact age of 2 years (IQR 28). The most frequent contact ages were 1 and 2 years with 24 (25%) and 23 (24%) recorded contacts, respectively. Ten contacts (10%) occurred with other infants aged less than 12 months.

### Across commune mixing

In this study 45% of the contacts occurred with persons living in a different commune from that of the infant. Eight of the 14 infants made contact with at least one person from a commune different from the commune where they reside. Of infants attending a day-care centre in a commune different from the commune in which the infants reside 86% made contact with person(s) from different communes, while 29% attending a day-care centre in their home commune did so. Infants cared for in a day-care centre with more children (86% of those in a centre with > 15 children vs 29% of those in a centre with < 14 children) and with more childcare personnel (100% of those in a centre with 4–12 childcare personnel vs 25% of those with two to three childcare personnel) on average had an elevated probability for contact with a person from a different commune. Additionally, four of the ten day-care centres recorded contacts with persons from more than one different commune: contacts with persons from two different communes in two centres, four different communes in one centre, and five different communes in another centre.

### Infant-infant contact

Ten percent of the contacts occurred with other infants younger than 12 months old. Half of the infants made contact with at least one other infant at day-care. Half of the enrolled day-care centres had more than one infant. Seven of nine infants in those day-care centres made contact with at least one other infant, while all infants attending the centres with only one infant (the subject only) did not have contact with other infants.

## Discussion

### Summary

This is the first study that focuses on the physical contact patterns of infants in day-care settings. The study included 14 participants who were in contact with 96 different individuals in day-care over the course of one day. Most contacts were with other children and 10% were with other infants. More contacts occurred for infants who could walk and, crucially, in groups with more children in care at day-care. Nearly 60% of the infants had across commune contacts in day-care, while half of the infants went to another commune to attend a day-care centre.

Infants who could walk by themselves tended to have more contacts during day-care. Our study implies that walking brings about higher activity to touch or to be touched more frequently with other individuals at day-care; however, almost all infants were already able to roll over, crawl, and sit themselves up, so we were unable to look at those activities as factors. Age could work as a confounder in an association between walking and contacts, and we adjusted RR by age group, while walking could be partially on the causal pathway from age to the infant’s contacts, and we saw only crude RR of age group on the mean number of contacts. In this study, at day-care, the infants’ age did not increase the rate of contacts very much, though the participants in this study were relatively similar in age (6–11 months). Moreover, infants with larger numbers of children in their group on average had more contacts, while neither room/facility size of day-care nor total number of children in a day-care centre was associated with elevated contact rates. We found that, unsurprisingly, infant contact rates scaled with the size of the child’s group in day-care and that cheaper day-care facilities had generally larger groups.

### Comparison to previous studies

The average number of contacts with different individuals was 6.9 (SD 3.2) during the infants’ stay at day-care on the day of observation. Previous studies have found the average number of contacts of an infant per day outside of day-care to range from 4.3 (Kovacs, personal communications) to 4.6 ^7^ or to 6.68 ^8^. However, these studies collected contact information by asking the mother/guardian to record all the contacts in one day. Skin-to-skin contacts as well as interactions in close proximity with three or more words directed to the infant were counted as contacts in the latter two studies, so the number of only physical contacts in these studies must have been lower than those shown in the studies as the number of contacts.

In this study, the majority of infant contacts at day-care occurred with other children who were cared for at the same day-care centre (63%) and with adults working there as childcare personnel (31%). These contacts were made on a daily or almost daily basis (84%). Contacts with other children mostly lasted only for a few minutes, and those with childcare personnel lasted longer (greater than five minutes in duration). Similarly, studies of infants outside of the day-care setting found most contacts to be regular ^8^; however, those contacts usually lasted over multiple hours (Kovacs, personal communications, ^7, 8^). This may be in part due to the difference in the definition of contact in this study and those in the previous studies ^7, 8^. For example, previous studies included both interaction in close proximity and physical contacts, which means they measured the length of time during which the persons simply spent time close together. In contrast, in this study, we precisely measured only the time during which a subject had skin-to-skin contact with another person by observation. The method used in this study is more likely to correlate with the transmission of *Streptococcus pneumoniae* because it is generally assumed to be transmitted through close interpersonal contact (i.e. skin-to-skin contact) ^9, 10^.

More than half of the infants made contact with at least one person from a commune different from their own commune at day-care. Four of the ten day-care centres recorded infant contacts with persons from more than one different commune. Half of the participants in this study attended a day-care centre beyond the borders of their commune of residence. This finding was surprising because education and health in Vietnam are both largely organized within administrative communes. It is probably because in Vietnam, privately-operated day-care centres are commonly chosen for infant care, and these are not related to the administrative area. Additionally, mothers may be likely to use day-care centres close to their workplace or their supporter’s residence (e.g. grandparent’s house) rather than their own residence. The infant contact study in Nha Trang, 2017 (Kovacs, personal communications) found that contacts outside of day-care were mostly localized within the same household and within the same commune, with only four percent of all the contacts occurring outside the infant’s commune. Generally, infants are likely to make contact with individuals in their own commune; however, those attending day-care could have many more opportunities to mix with people outside their own commune. Thus, day-care could be a means to accelerate the spatial spread of childhood infections.

The age of contacts ranged from 0 to 65 years, skewed to young children, with a median contact age of 2 years. The most frequent contact age was between 0 and 2 years following the age of people who were cared for at day-care. This is different from the age distribution of infant contacts in previous studies (Kovacs, personal communications, ^7, 8^), in which adults aged 20–40, consistent with the parent’s age, were major contributors. A peak in adults was lower and wider in this study following the age distribution of childcare personnel. Previous studies have also shown another peak in the age groups of 0–5 (Kovacs, personal communication, ^7^) or 0–4 ^8^ years, but the proportion of contacts aged 0–5 at day-care in this study (63%) was much higher than that in those studies (12%—age group 0–5 in Kovacs’s study, and 8%—age group 0-5 in Oguz *et al*.’s study ^7^). In particular, the proportion of contacts with other infants aged < 12 months was remarkably higher (10%) than that recorded in the Nha Trang study in 2017 (5 out of 430, recorded as part of group contact). Infant contacts with other infants do not seem to occur very often in general but commonly occur in the day-care setting, especially if there are higher numbers of infants in the same day-care group.

### Limitations

In Vietnam, it is not common to use day-care for children aged less than 12 months. Families typically use privately paid-for day-care if needed because they have more space to care for infants. Small, private childcare locations are likely not registered with the relevant administrative units, and hence, we are likely to have not identified all day-care options for infants in Nha Trang. Nevertheless, this study selected day-care centres to be largely representative of the whole Nha Trang area.

Our study enrolled 14 infants because the primary endpoint of interest, in the absence of any other data to inform such, was a reasonably precise estimate of the number of contacts in a day-care setting. This did not allow for much statistical power to investigate in detail which characteristics are associated with the number of infant contacts. Therefore, our analyses are largely descriptive. Future studies may wish to investigate infant contacts on a larger scale to determine environmental factors that may influence the spread of *Streptococcus pneumoniae*.

## Conclusions

This study found that day-care attendance may be one factor that increases the contact rates of infants in Nha Trang and diversifies those contacts in terms of age and geographical spread; i.e. nursery attendance leading to more contacts with young children and across the borders of communes. In this study, day-care attendance not only increased contact rates beyond those usually experienced by young children cared for at home, seen in the previous studies, but also specifically increased the contact rates with other children and adults from other communes. Day-care may play a key role in the transmission of respiratory pathogens like *Streptococcus pneumoniae* to infants. In addition, we found that infants who could walk by themselves and who with larger numbers of children in their group at day-care had more contacts. It also may be a specific area that should be investigated more closely to potentially help control of the spread of the pathogens in day-care management.

## Data availability

### Underlying data

Open Science Framework: Infant contact in day-care centres in Vietnam: A cross-sectional study to understand infant infection risk. https://doi.org/10.17605/OSF.IO/YH468 ^13^

This project contains the following underlying data:

- infant_daycare_data.csv (Data of infants)
- daycare centre_daycare_data.csv (Data of day-care centres)
- contact_daycare_data.csv (Data of contacts)

### Extended data

Open Science Framework: Infant contact in day-care centres in Vietnam: A cross-sectional study to understand infant infection risk. https://doi.org/10.17605/OSF.IO/YH468 ^13^

This project contains the following extended data:

- Form1_contact information.pdf (Form used to record infant contact)
- Form2_daycare information.pdf (Form used to record day-care characteristics)

### Reporting guidelines

STROBE checklist for ‘Infant contact in day-care centres in Vietnam: A cross-sectional study to understand infant infection risk’. https://doi.org/10.17605/OSF.IO/YH468 ^13^
