# Supplementary material for: Infant contact in day-care centres in Vietnam: A cross-sectional study to understand infant infection risk
Source: Wellcome Open Res. 2019 Jun 11;4:75. Originally published 2019 May 1. [Version 2] doi: 10.12688/wellcomeopenres.15238.2 (PMC6544129; doi:10.12688/wellcomeopenres.15238.2)
Supplement: Peer review history

# Peer review: Reviewer response for version 1

**DOI:** 10.21956/wellcomeopenres.16631.r35421

**Published:** 2019-05-28

**Authors:** Melahat Melek Oğuz

**Affiliations:** 1 Department of Pediatrics, Dr. Sami Ulus Maternity and Children’s Health and Diseases Training and Research Hospital, Ankara, Turkey

I suggest some minor revisions to your manuscript.  

 The paper presents the analysis of data gathered in Vietnam on social contact patterns of infants in day-care centers. The work fits within the increasing literature on social mixing patterns relevant for infections transmission and covers an age strata on which up to today not much evidence has been gathered. Although the sample size is too small the work is interesting. However, I believe there is space for considerable improvement and, in particular, the inclusion of a more extensive explanation of the study design and a more thorough analysis of the data gathered. 

 Particular comments on the work include the following: 

1. Abstract section: In methods; line 4 use “factosr that associted with contact numbers” instead of “its factors”. Conclusion could be made more specific for the current study. You can not say the day care center attendance one factor that increase contact rates of infants. Because in this study there is not any control group who has not attended day-care center
2. Introduction: This section is rather clear and to the point, goals of current study in addition to what is available should be added.
3. Methods section: How was the day of the interview selected?  
4. Can the authors provide more details about the participants and their representatives? 
5. Discussion: This section is clear.
6. Conclusion: Conclusion could be made more specific for the current study. You can not say the day care center attendance one factor that increase contact rates of infants. Because in this study there is not any control group who has not attending day-care center. How can you be sure about this?

I have read this submission. I believe that I have an appropriate level of expertise to confirm that it is of an acceptable scientific standard.

# Response

**Published:** 2019-06-03

**Authors:** Michiko Toizumi

**Affiliations:** Institute of Tropical Medicine, Nagasaki University, Japan

Dear Dr. Melahat Melek Oğuz,

Thank you very much for your valuable comments. We revised the manuscript following the reviewer’s suggestions. 

   

- 1. Abstract section: In methods; line 4 use “factosr that associted with contact numbers” instead of “its factors”. Conclusion could be made more specific for the current study. You can not say the day care center attendance one factor that increase contact rates of infants. Because in this study there is not any control group who has not attended day-care center

Thank you for your suggestions. We replaced “its factors” to “factors that associated with contact numbers”. You are right, we cannot say that day-care attendance increased contact rates only by this study. We added some words in Discussion section to state that this was from discussion comparing with contact rates at home in previous studies: “In this study, day-care attendance not only increased contact rates beyond those usually experienced by young children cared for at home, seen in previous studies,”

- 2. Introduction: This section is rather clear and to the point, goals of current study in addition to what is available should be added.

 

We stated: “This study aimed to describe the frequency and nature of infants’ physical contacts, as close interpersonal contacts relevant to the transmission of Streptococcus pneumoniae (9, 10), in the day-care setting to aid our understanding of infant infection risk stemming from these settings in Nha Trang, Vietnam.” We added more detail information of the study setting to show what is available there in Method section: “Nha Trang is a tropical coastal city with a mix of high income through tourism and poorer rural areas (11). Mean number of family members in a household was 5.4 (standard deviation 2.1) in a community survey targeting children under two, in Nha Trang, 2016 (12). In that survey, 6% of infants aged <12 months and 46% of children aged 12-23 months were attending day-care centres or nurseries.”

- 3. Methods section: How was the day of the interview selected? 

 

We stated: “The days were chosen at the observers’ convenience. All were weekdays; seven observations were conducted on Tuesdays, five on Wednesdays, and two on Thursdays.”

- 4. Can the authors provide more details about the participants and their representatives? 

We mentioned the participants’ representativeness as below. In Method section, we explained how we selected them across the whole study area: “We randomly selected 10 Nha Trang communes with at least one infant attending a day-care centre under the following criteria that ensured a spread of day-care centres across the whole study area: 1) for each of the five arms of an ongoing cluster randomised PCV trial in the Nha Trang area, in which all 27 communes in Nha Trang were randomly divided into five arms (12) , 2) randomly select one rural and one urban commune with at least one infant attending a day-care centre, 3) randomly select one day-care centre per selected commune, and 4) randomly select up to three infants per day-care centre for enrolment.”. And in Discussion section, we explained that we could not figure out the study population but tried selecting our study samples from day-care centres largely representative of the whole study area: “Small, private childcare locations are likely not registered with the relevant administrative units, and hence, we are likely to have not identified all day-care options for infants in Nha Trang. Nevertheless, this study selected day-care centres to be largely representative of the whole Nha Trang area.”

- 6.  Conclusion: Conclusion could be made more specific for the current study. You can not say the day care center attendance one factor that increase contact rates of infants. Because in this study there is not any control group who has not attending day-care center. How can you be sure about this?

Thank you for the important suggestion. You are right, we cannot say that day-care attendance increased contact rates only by this study. We added some words to state that this was from discussion comparing with contact rates at home in previous studies: “In this study, day-care attendance not only increased contact rates beyond those usually experienced by young children cared for at home, seen in previous studies,”. Also, to be more specific for the current study, we stressed our findings in the conclusion: “In addition, we found that infants who could walk by themselves and who with larger numbers of children in their group at day-care had more contacts. It also may be a specific area that should be investigated more closely to potentially help control of the spread of the pathogens in day-care management”.

# Peer review: Reviewer response for version 1

**DOI:** 10.21956/wellcomeopenres.16631.r35420

**Published:** 2019-05-20

**Authors:** Alessia Melegaro

**Affiliations:** 1 “Carlo F. Dondena” Centre for Research on Social Dynamics and Public Policies, Bocconi University, Milan, Italy

Toizumi and colleagues investigate on a very important question that has not been yet addressed so specifically in other works. Indeed they gather data to describe infants mixing interactions in a day care setting. This kind of information is critical to better understand diffusion processes of childhood infectious diseases and to evaluate the impact of alternative control measures.

   

 The work is nicely presented and clear. The main limitation is the number of children enrolled in the study (14 infants) which make any conclusion very specific to the sample and the day care settings being evaluated. This said, however, I think the work is of interest to the infectious disease community and should be accepted. 

 I think more effort should be put into better describing how contacts are defined here and who is measuring these. In the paper it says "staff" but I imagine that with infants it is not so easy to really quantify the precise number of interactions. In addition, it seems that it is not the day care size (sqm and people) that matters but rather the infant's group. Bigger group report double the number of contacts as opposed to smaller ones. This aspect is extremely relevant for infections transmission and it should be further stressed in the text.

 In Table 2 rather than the gender of the contact I would evaluate the assortativeness by gender if anything.. 

 Results: Is the kind and number of contacts changing for infants that are attending more/less frequently the day care center?

More specific comments:

 Abstract is vague in the method. What kind of setting is being evaluated?

 Results: From Infants...to day-care should be made clearer..

 How are commune defined by the way? What is a commune in this setting? More details on the setting in the main text should be included

 Conclusions: ...diversifies them in terms of age and geographical spread...

 What are the authors referring to here?

 Introduction:

 Reduced dose OF PCV schedule

 Methods:

 One to three infants...were enrolled...

 How is this decided? Whether one or three?

 Staff from...observed...

 Staff meaning? How did they observe and record?

 Results:

 6 other contacts in Table 2, whereas it is only 5 other contacts in the text. check.

I have read this submission. I believe that I have an appropriate level of expertise to confirm that it is of an acceptable scientific standard.

# Response

**Published:** 2019-06-03

**Authors:** Michiko Toizumi

**Affiliations:** Institute of Tropical Medicine, Nagasaki University, Japan

Dear Dr. Alessia Melegaro,

Thank you very much for your valuable comments. We revised the manuscript following the reviewer’s suggestions.

 

- I think more effort should be put into better describing how contacts are defined here and who is measuring these. In the paper it says "staff" but I imagine that with infants it is not so easy to really quantify the precise number of interactions. In addition, it seems that it is not the day care size (sqm and people) that matters but rather the infant's group. Bigger group report double the number of contacts as opposed to smaller ones. This aspect is extremely relevant for infections transmission and it should be further stressed in the text.

Thank you for your suggestion. We changed the word “Staff” to “trained field workers” to clarify that the observers and the childcare staff were independent. We described how they observed and recorded more specifically: “First, the observers chronologically noted each physical contact with the name of the contact person and the precisely measured time of the contact. Secondly, they added up the times of the contacts by the contact person and recorded the contacts using the contact record.” To stress that infants cared for in a larger group had more contacts at day-care, we added a sentence as below at the end of conclusion: “In addition, we found that infants who could walk by themselves and who with larger numbers of children in their group at day-care had more contacts. It also may be a specific area that should be investigated more closely to potentially help control of the spread of the pathogens in day-care management.”

- In Table 2 rather than the gender of the contact I would evaluate the assortativeness by gender if anything..  

Thank you for your suggestion. We added “assortativeness by gender” in Table 2 and a sentence in the text: “It also could been seen in less assortative gender contacts among childcare personnel.”

- Results: Is the kind and number of contacts changing for infants that are attending more/less frequently the day care center?

We added “frequency of attending day-care” in Table 1 and in the text. There was no association between number of contacts and the frequency. There was only one infant attended day-care twice a week and the other 13 did almost daily (5-7/week) so it is difficult to discuss the associations.

- More specific comments
- Abstract is vague in the method. What kind of setting is being evaluated? 

We added more information of the setting in the Method: “Nha Trang is a tropical coastal city with a mix of high income through tourism and poorer rural areas (11). Mean number of family members in a household was 5.4 (standard deviation 2.1) in a community survey targeting children under two, in Nha Trang, 2016 (12). In that survey, 6% of infants aged <12 months and 46% of children aged 12-23 months were attending day-care centres or nurseries”. 

- Results: From Infants...to day-care should be made clearer..
- How are commune defined by the way? What is a commune in this setting? More details on the setting in the main text should be included

Thank you for your suggestion. We added a sentence in Methods to explain “commune” in this setting: “In Vietnam, a commune is the smallest municipal administrative unit, and each commune has one commune health centre providing basic healthcare services for the community including vaccination. Nha Trang City comprises 27 communes.” 

 

- Conclusions: ...diversifies them in terms of age and geographical spread...
- What are the authors referring to here?

 

We are referring to more contacts with young children and more contacts across the borders of communes at day-care. We added: “diversifies them in terms of age and geographical spread; i.e. nursery attendance leading to more contacts with young children and across the borders of communes”.

- Introduction:
- Reduced dose OF PCV schedule

Thank you. We added “of” in the part.

- Methods:
- One to three infants...were enrolled...
- How is this decided? Whether one or three?
- Staff from...observed...
- Staff meaning? How did they observe and record?

The number was decided because we thought that an observer could closely observe up to three infants at the same time. This reason was added to the text: “because we felt that three was the maximum number of infants an observer could closely observe at the same once”. We changed the word “staff” to “trained field workers” and described how they observed and recorded more specifically: “First, the observers chronologically noted each physical contact with name of the contact person and time of the contact measured precisely. Secondly, they summed the time of the contacts by the contact person and recorded the contact using the contact record.

- Results:
- 6 other contacts in Table 2, whereas it is only 5 other contacts in the text. check.

Thank you for pointing it out. We corrected the text as: “The remaining contacts were with the family of childcare personnel (n = 3), other children’s parents (n = 1), and one mother and one father who visited her/his child in the middle of day-care (n = 2)”.
